# Reviews

**Published:** 1885-01

**Authors:** 


### REVIEWS.

A TEXT BOOK OF OPERATIVE VETERINARY SURGERY. By George Fleming, LL.D., F.R.C.V.S., with numerous illustrations. London: Ballière, Tindall & Cox, 20 King William street, Strand. New York: William R. Jenkins. Part I, pp. 266.

Anything from the pen of the well-known editor of the *Veterinary Journal*, particularly when dealing with an at once practically important and yet neglected topic, is certain to come before a large class of readers. For this reason it might be expected that we would devote some space to pointing out its good features, expatiate on the debatable ones, and criticize the faults and omissions if there be any. But really there is little room, either for special encomiums or censure. The plan of the work is simple and its scope limited, and the author does not claim to have exhaustively treated the subject. That in nearly every scientific respect the book is inferior to the continental treaties of DEGIVE, HERING, and TOUSSAINT-PEUCH, is evident on superficial examination; but it would be difficult to find anything superior to it in the English language. Its excellences are such as characterize English literary productions as contrasted with the German. The latter are, if more elaborate and philosophical, also more obscure, involved and sententious. Fleming's work is clear, written in a fair style, and shows that its author possesses admirable facilities for condensing matters for the student. It is not a very flattering reflection, regarding the *status* of Anglo-Saxon veterinary medicine, but it is not the less a true one, that these very qualities guarantee the success of the book. It is just the thing the American student requires.

In speaking of the general preliminaries of operations on animals, such as the method of securing them, the author exhibits a humanity which is in pleasant contrast with the jockey spirit so often assumed and then developed into an unconscious habit, by those who seek to cover their ignorance or inexperience under a show of *brusque* self-confidence.

The description of the methods for "throwing" horses is brief and complete, and Professor Fleming demonstrates his largeness of mind by incorporating descriptions of Farmer Miles' method, who, although an American, was abused and libelled by a domestic clique, and compelled to cross the Atlantic to secure recognition of his new and valuable additions to surgical procedure. The section on the dangers to which horses are liable when thrown, is bodily extracted from Professor Dieckerhoff's authoritative article on the subject.

We do not feel inclined to be finical, and the fault we are about to refer to —if it be so regarded by others—is a common one in human and veterinary surgical text books, but is it not really high time that wood cuts illustrating how the knife is to be held and text detailing it, be banished to make room for more necessary and useful material? Do not let us be misunderstood here! To hold a knife correctly is the very letter A of surgery, but the man who does not instinctively hold his knife right, or who has not learned how to use it in the dissecting room, has mistaken his profession, and all the wood cuts in the world will not help him. Then, it is impossible in a textbook to illustrate all the devices which the surgeon is called on to make on the spur of the moment.

As far as we can judge from the fragment of the work before us, its great defect is in the field of topographical (that is true surgical) anatomy. The anatomical descriptions and drawings are acknowledged to be taken from the author's translation of CHAUVEAU. Now, Chauveau is an excellent anatomical treatise, but it deals rather with systemic and comparative than with surgical anatomy. Professor Fleming would have found in the German Atlanti much better models and such as are suited for his purpose. We regret that he has not shown more independence of precedent here.

We await the second part of the work with interest. The publishers have done their part of the work well. E. C. S.

THE ART OF TAMING AND EDUCATING HORSES. By D. Magner. Pp. 1088; with 900 illustrations. Battle Creek, Mich.: Review & Herald Publishing House. 1884.

This ponderous—and some hypercritical persons might say nondescript—volume is evidently the product of gradual evolution. The intentions of the author were evidently excellent, and he has succeeded in carrying them out fairly well in some directions ; in others he has hampered himself considerably by exercising less discretion and good taste than his best friends would wish him to have shown.

It seems that Mr.—or, as he styles himself (on the same principle that the washerwoman in Nicholas Nickleby might have styled herself a professor of the lavatory art, because she "professed" to use a mangle), Professor—D. Magner has practised for many years a method of taming wild and vicious horses as a secret; that he has been in the habit of teaching this method to classes formed in every part of the Union, as a secret, and that now he has concluded to make the said secret public. So far so good. No one will censure Prof. Magner for thoroughly reaping the financial harvest first, and then establishing his claims to literary priority by killing the goose when she would lay no more golden eggs.

In addition to publishing his method of taming horses, he publishes chapters on shoeing, diseases of the foot, and the more common surgical and internal diseases of the horse. These chapters of the book are in part the work of three well known veterinary surgeons, formerly editors of the *Veterinary Gazette*, Drs. Hamill, Meyer, and McLaughlin.

Composed of such heterogeneous material, and being the work of so many hands, this book exhibits many shades of merit. The work of Prof. Magner's veterinary associates is of a higher order than the rest of the book; that on shoeing, for example, contains citations from Hamill which have not been elsewhere published, and some from Prof. Fleming, which conjointly constitute as thorough a disquisition on the subject as we are acquainted with. The illustrations of this part are very elaborate, and Prof. Magner has evidently spared no expense in making them as attractive and true to nature as possible. The chapter on the diseases of the foot is equally well written, and nearly as well illustrated. That on the physiology of the circulation, which introduces the subject of internal diseases generally, is also up to the times. A strange error mars the upper figure reference on page 740, where the natural pigment corpuscles of the skin of a frog's web, are named "spots of congestion," produced by "alcohol."

The division of the work which bears the impress of Magner's original work relates to the method of subjugating horses alluded to in the opening, and includes an account of over forty cases of wild, vicious, or stupid horses which he has tamed, and interspersed here and there are illustrations showing how the horse looked "before taking" and "after taking"—somewhat on the principle of "Wolcott's Pain Annihilator," "Laird's Bloom of Youth," etc. The reader will recollect advertisements in which two faces are shown, the first with sunken cheeks, a bald head, villanous leer, disgusting secretions running from every cavity, and pustules "all over"; the second represents an attractive Venus, who was supposed to be metamorphosed from the disgusting creature shown in the first picture, by the use of the magic remedy advertised. Prof. Magner's woodcuts, in like manner, exhibit the forty horses, rearing, kicking, plunging, eating people, jumping over hay-carts, etc., in the first act, and quietly carrying Prof. Magner, pulling Prof. Magner, looking at Prof. Magner with fond eyes, or cringing before his stare, in the second act. Prof. Magner does not inform us whether these cuts are from instantaneous photographs or not. If they are, they might have some convincing value. As it stands, they seem to have none.

Numerous testimonials from horsemen, clubs, and clippings from newspapers, lauding Prof. Magner's achievements, are strewn through these chapters, evidencing that the great horse-tamer is not inclined to hide his light under a bushel.

Briefly, Magner's methods are three: the first consists of throwing the horse, the second is bringing his head and tail close together and keeping them fixed, the third, and to us the most novel as well as debatable method, is the production of pressure on the medulla oblongata, through the soft parts overlying the membrana obtruatoria. We will inform Professor Magner that it is not likely that he succeeds in accomplishing what he thinks to be the object of this method. It is not as easy as he seems to believe to effect pressure on so deep an organ except by altogether inadmissable means. The real action of this method seems to be on the same principle as the subjugation of the horse when castrated standing, by producing a powerful impression on the peripheral nervous system. Should he really effect such phenomena as making the horse " look as if he were going to sleep, pant and break out in perspiration," he would have produced an effect on the nerve centres or the pneumogastric nerves which it would not be safe to prolong or to repeat. In that event we should hear of blind staggers as a not infrequent result of the application of the pressure method.

The principles announced as governing the methods of subjection are excellent, and the illustrations of the different dispositions of horses are as good as the woodcutter's art can render them. We object, however, to the pictures of human beings, illustrating dispositions in horses, and which are really nothing more nor less than temperance sermons in disguise. The only human picture that is at all relevant, is the frontispiece representing Prof. Magner himself, who would doubtless attain his ardent ambition of being considered a great man much better if he had had self-denial enough to suppress this thing. His picture represents him as a fair-looking, good-natured and shrewd man, and we are happy to make his acquaintance *in figure*, but we know of many who will cavil at it.

In his title the author announces recipes for remedies which had long been sold as secrets. Those published by him in the back of the volume are, as we use to say during the war, not worth a continental shinplaster. In justice to his able and accomplished co-workers, Prof. Magner should have omitted this discreditable addition, even if he had not recognized its impropriety on every other ground. There is also a too palpable tone of advertisement throughout the volume which, in some places, goes far beyond the limits of good taste.

Excluding the features above adverted to, and which for many of his readers will not constitute serious disadvantages, we think that we can cordially recommend it to horsemen, farmers, stock-breeders and others interested in the management of the horse, as containing many sound reflections, valuable hints and interesting facts. It constitutes a good common-sense, domestic reference book for those who have a stable or who are compelled to handle horses. E. C. S.

Quarterly Report of Veterinary Progress. By J. H. Steel and F Smith. April, May, June, 1884.

This little pamphlet devotes its twenty-eight pages to the recent advances made in medicine and surgery, in so far as they are of interest to the veterinarian, giving a synopsis of the principal veterinary and medical periodicals published at the present time, and placing before the reader a concise statement of the work performed in the last three months by our profession throughout the world.

Anthrax, glanders, rabies, tuberculosis and scarlatina receive special attention, and the labors of Pasteur, Koch, Chauveau, Bouley, Billings, Stickler and Law are referred to.

Extracts from the French, German and Italian journals are translated. To the veterinarian the pamphlet will be invaluable, giving him, as it does, a clear summary of what otherwise could only be obtained by the careful poring over many hundred pages of periodicals, and saving him the great expense incurred therein.

CONTAGIOUS DISEASES (ANIMAL) ACT, 1878, Dublin, Ireland.

The report of the Council for the year ending 1882 contains the provisions of the Act and Statistics, showing the results of contagious or infectious diseases as per return of local authorities.

The Act defines the duties of the authorities and veterinary surgeons in each disease separately, giving comprehensive directions as to notification, movement into and out of infected districts, slaughter, compensation, disinfection, quarantine, etc.

The Report also contains a number of forms for the use of Inspectors, declarations, licenses, etc., which will be of value to Veterinarians.

ANNALS OF SURGERY. A Monthly Journal devoted to Surgical Science and Practice. Edited by L. S. Pitcher, M.D., Brooklyn, N. Y., and C. B. Keitly, F.R.C.S., London, Eng. 1885.

The publishers announce that the first number of this journal will be issued this month. Judging from the high character of its predecessor, the "Annals of Anatomy and Surgery," we predict for our readers a scientific treat in the appearance of this journal.

The price will be $5 a year, and 50 cents a number.

### BOOKS AND PAMPHLETS RECEIVED.

R. SCUOLA, SUPERIORE DI MEDICINA VETERINARIA DI MILANO. Milan, 1884.

EXPLANATION OF THE PATHOLOGY AND THERAPEUTICS OF THE DISEASES OF THE NERVE-CENTRES. By I. McF. Gaston, M.D. Atlanta, Ga., 1884.

THE DRY TREATMENT OF CHRONIC SUPPURATIVE INFLAMMATION OF THE MIDDLE EAR. By Prof. Charles J. Lundy, M.D. Detroit, 1884.

MURIATE OF COCAINE IN OPHTHALMIC SURGERY. By Charles J. Lundy, M.D. Detroit, 1884.

PHYSICIANS' VISITING LIST FOR 1885. Published by P.Blakiston, Son & Co., Philadelphia.

REPORT OF THE BROOKLYN COMMISSIONER OF HEALTH, December, 1884.

JOHNS HOPKINS UNIVERSITY CIRCULARS.

ANNALS ET BULLETIN DE LA SOCIÉTÉ DE MEDICINE DE GAND.

JOURNALS.—La Clinica Veterinaria, Milano. The Veterinarian, London; The Veterinary Journal, London; Der Zoologische Garten, Frankfort; Schweizerisches Archiv fur Thierheilkunde, Zurich; Archiv fur Wissenschaftliche und Praktische Thierheilkunde, Berlin; Wochenschrift fur Thierheilkunde und Viehzucht, Augsburg; Tidskrift for Veterarin-Medicin, Stockholm; Journal de la Société contre L'Abus du Tabac, Paris; Giornale di Anatomia, Fisiologia e Patologia, degli Animali, Pisa; La Cronica Medica, Valencia; Kansas City Review of Science and Industry; The College and Clinical Record, Philadelphia; New England Medical Monthly, Sandy Hook; Nashville Journal of Medicine and Surgery; The Southern Clinic, Richmond; The Western Medical Reporter, Chicago; Journal of Cutaneous and Venereal Diseases, New York; Denver Medical Times; The St. Joseph Medical Herald; The Weekly Medical Review, St. Louis; The Blacksmith and Wheelwright, New York; National Live Stock Journal, Chicago; The Breeder's Gazette, Chicago; Cultivator and Country Gentleman, Albany; Weekly Drover's Journal, Chicago; The Chicago Tribune; Horseshoer and Hardware Journal, Chicago; The Polyclinic, Philadelphia; Medical World, Philadelphia; American Veterinary Review, New York; Atlantic Journal of Medicine, Richmond; Indiana Farmer, Indianapolis; Texas Courier Record, Fort Worth; The Jersey Bulletin, Indianapolis; American Journal of Ophthalmology, St. Louis; Pacific Medical and Surgical Journal and Western Lancet, San Francisco; New York Medical Journal; The Quarterly Journal of Veterinary Science in India; Hearth and Home, Washington; American Liveryman and Horseowner, Chicago; American Psychological Journal, Philadelphia.

